# Communicating Antimicrobial Resistance on Instagram: Content Analysis of #AntibioticResistance

**DOI:** 10.2196/67825

**Published:** 2025-08-20

**Authors:** Elin Nilsson, Emma Oljans, Anna-Carin Nordvall, Mirko Ancillotti

**Affiliations:** 1 Umeå School of Business, Economics, and Statistics, Umeå University, Handelshögskolan, Biblioteksgränd 6, Umeå, 90187, Sweden, 46 730612007; 2 CERUM (Center for Regional Science), Umeå University, Umeå, Sweden; 3 Department of Women’s and Children’s Health, Uppsala University, Uppsala, Sweden; 4 Department of Movement, Culture and Society, Swedish School of Sport and Health Sciences, Stockholm, Sweden; 5 Department of Business Studies, Uppsala University, Uppsala, Sweden; 6 Centre for Research Ethics and Bioethics, Department of Public Health and Caring Sciences, Uppsala University, Uppsala, Sweden

**Keywords:** antimicrobial resistance, social media engagement, communication, Instagram, health communication, content analysis

## Abstract

**Background:**

Antimicrobial resistance (AMR) is a major global health issue heavily influenced by human behavior. Effective communication and awareness-raising are crucial in curbing AMR, with social network sites (SNSs) significantly shaping health behaviors. Despite their potential, current analyses of AMR on SNSs have focused mainly on top-down communication initiatives.

**Objective:**

This study aims to examine AMR on Instagram (Meta Platforms), identifying key actors, content themes, and the nature of the communication to understand how AMR is portrayed and perceived.

**Methods:**

Based on the sender-message-channel-receiver model, this study used content analysis to review publicly accessible posts on Instagram. The data refer to 24 months, focusing on the hashtag “#antibioticresistance.” After cleaning the data, 610 posts (10% of the total 6105) were analyzed.

**Results:**

Content creators were predominantly information drivers or professionals in science and health. Posts frequently featured text-dominated visuals or images of bacteria and laboratory tests. However, the AMR posts were found to be siloed, with limited engagement beyond specific interest groups. The study highlighted the neutrality and accuracy of the content but noted the challenge of reaching a broader audience.

**Conclusions:**

While Instagram serves as a platform for accurate and informative AMR communication, the post of it remains confined to niche groups, limiting its broader impact. To enhance engagement, AMR discussions should be integrated into more general interest content, use visually compelling formats, and encourage institutional participation and interactive user engagement.

## Introduction

### Background

Antimicrobial resistance (AMR) is a major global issue, causing substantial health burdens and societal costs. Every year, about 1.3 million people die from infections caused by antimicrobial-resistant bacteria, and this figure is predicted to increase to 10 million by 2050 [1,2].

### The Role of Community Awareness in Combating AMR

Community knowledge and awareness raising are key factors in curbing resistance because AMR largely depends on human behavior [3-5]. In addition to medical use, which is the primary driver of AMR [6,7], there is plenty of individually and contextually induced health and lifestyle behavior impacting human health—but also animal health and the environment—such as food production and consumption [8], international travel [9], or the adoption of basic hygiene practices to avoid infection spread [10]. Previous research stresses the necessity of considering the contextual motivations and preferences of participants and that health communication needs to explore and engage with these [11,12]. The World Health Organization’s (WHO’s) Global Action Plan highlights the need to emphasize and improve AMR awareness and understanding through effective communication, education, and training [13].

### The Influence of Social Network Sites on Health Behaviors

Social influences shape health and lifestyle behaviors to a high degree. Research has highlighted the influence exerted by social network sites (SNSs), which stand as structural determinants of health nowadays for their role in information exchange and diffusion of beliefs [14]. SNSs are web-based services that allow individuals to (1) construct a public or semipublic profile within a bounded system, (2) articulate a list of other users with whom they share a connection, and (3) view and traverse their list of connections and those made by others within the system [15]. SNSs are frequently used to address and communicate health issues [16]. They were used in a few intervention studies to raise awareness of AMR, for educational purposes, and to promote behavioral change [17-21]. International health bodies such as the WHO and the European Centre for Disease Prevention and Control also engage in SNSs—primarily during Antimicrobial Awareness Weeks—and promote their use in awareness-raising campaigns [22,23]. However, to date, the analysis of AMR on SNSs has been almost completely limited to top-down communication, with a primary focus on X (formerly Twitter) [24-26]. Recent studies have highlighted the effectiveness of SNSs in rapidly disseminating health-related evidence to professionals, particularly during public health emergencies, where timeliness and platform dynamics influence uptake and visibility [27]. At the same time, SNSs demonstrated considerable potential for reaching broader audiences with public health messages, influencing awareness, attitudes, and behaviors [28]. However, the very features that facilitate wide dissemination can also contribute to the amplification of misinformation. A systematic review found that a significant proportion of health content on popular SNSs is inaccurate or misleading, especially in emotionally charged or polarizing contexts [29]. A recent bibliometric study showed a sharp rise in research on how SNSs spread health misinformation during the COVID-19 pandemic, underlining the need for active monitoring and strategies to reduce harm [30]. Content analyses of platforms such as YouTube have also revealed large amounts of misleading health information, such as false claims about vitamin D as a treatment for COVID-19, highlighting the difficulties in ensuring reliable health communication online [31]. This dual potential underscores the importance of developing communication strategies that not only ensure the visibility of accurate and accessible information but also address the risks associated with misinformation, particularly in the context of AMR, where public understanding and behavior are crucial. It has been shown that institutional awareness events, such as the World Antimicrobial Resistance Awareness Week (WAAW)—the major global initiative dedicated to raising awareness and promoting action on AMR—can rapidly increase the number of posts, including among laypeople, although this increase tends to be short-lived, typically returning to baseline levels within 48 hours [25]. Another study focusing on Twitter looked into the types of influential users and showed that the discussion was primarily influenced by news sources, health professionals, and governmental health organizations [24]. A study analyzing the information shared by Instagram (Meta Platforms) users of oral and topical antibiotics for treating acne vulgaris claimed the potential use of the SNS in elucidating patients’ behavior and attitudes [32].

Among SNSs, Instagram is a platform in steady growth [33], among the most popular in size (fourth SNS globally, with 1478 million active users in 2022) [34] and impact on users’ decision-making [35]. Content-wise, Instagram is a favorite SNS for social purposes [36]; therefore, it holds the potential for spreading knowledge and raising awareness of AMR, or on the contrary, to contribute to misinformation. Furthermore, considering that Instagram is an image-based platform of the first choice for entertainment purposes and co-creating with brands via social media [36], where topics such as food, wellness, and travel are amongst the most typical, the influence on health and lifestyle behavior of relevance for AMR is also worth attention.

To explore how Instagram content may shape awareness and behaviors related to AMR, it is useful to apply a theoretical model that captures the dynamics of message dissemination and reception.

### Understanding Berlo’s Sender-Message-Channel-Receiver Communication Model

The sender-message-channel-receiver (SMCR) communication model, developed by Berlo in 1960 [37,38], outlines 4 essential components in the communication process: the sender, the message, the channel, and the receiver. This model has been extensively used in communication research, including as a framework for understanding how SNS users disseminate and receive information [39-41].

The sender refers to the originator of the communication, whose characteristics, such as communication skills, knowledge, and attitudes, influence the effectiveness of the message delivered. In SNS contexts, the sender is any individual or organization posting content, with their social status or credibility impacting how their message is perceived.

The message itself consists of the information being transmitted. It can take various forms, including text, images, or videos, depending on the channel used.

The channel is the medium through which the message is transmitted, such as visual or auditory platforms. On Instagram, which serves as the primary communication channel in this context, users interact primarily through visual and textual content. In addition, online communities can also be considered vehicles or channels for displaying information. Typically, hashtags are not only part of the message but have a broad spectrum of functionality, including being a powerful tool for marketing promotion of products and services, acting as an evaluation marker that can set the interpretation model of the message, and activating networks of associations [42]. Finally, the receiver is the target audience of the message, whose characteristics—such as attitudes, social background, and previous knowledge—play a crucial role in determining how the message is interpreted.

### Studying AMR Communication on Instagram

This study aims to examine the communication about AMR on Instagram, identifying key actors, content themes, and the nature of the communication to understand how AMR is portrayed and perceived using Berlo’s SMCR communication model. Thus, the study contributes to addressing a broader challenge: while AMR is recognized as a pressing global health threat, little is known about how it circulates on SNSs like Instagram or how effectively these messages engage the general public. By analyzing how messages are framed, who produces them, and which publics they appear to reach, this study offers insights that can inform the development of more inclusive and effective digital communication strategies in the AMR field.

## Methods

### Study Design

This research uses a descriptive design and is grounded in inductive reasoning. The method of data collection and analysis is content analysis, which, in this study, integrates both quantitative and qualitative approaches [43,44]. The study incorporates quantitative elements but is primarily qualitative, guiding the choice to pursue trustworthiness as a measure of quality [45,46]. To foster transferability, a detailed description of the Methods and Results is offered. The study was planned following Bengtsson’s recommendations to enhance the credibility of content analysis [47]. In the planning phase, the scope, relevance, and breadth of the aim, the size and characteristics of the sample and unit of analysis, the choice of data collection method, the choice of analysis method, and the ethical implications were thoroughly considered.

### Data Collection

The data refers to the 24 months between December 1, 2020, and November 30, 2022. The timeframe was selected to include twice the WAAW, taking place in November. The feed posts (reels and stories were not considered) were collected retrospectively using an automated scripting tool for web scraping [48]. It was decided to use “#antibioticresistance” in the search, a widely used and recommended hashtag to engage in conversations about AMR [49], because it was deemed as a simpler but still scientifically proper expression of the notion, to which the public is relatively more used with respect to alternatives such as “antimicrobial resistance” or “AMR” [50].

The search returned 7380 hits. After cleaning the data, there were 6105 posts. Posts were excluded according to the following criteria: when non-English languages dominated the posts, duplicates, and reposts. To ensure the representativeness of the sample, hence enhancing the credibility and transferability of the study, a 10% sample ratio was applied (N=610). To prevent over-reporting content generated in concomitance with special AMR-related events, stratified random sampling was applied. For each month, 10% of the posts were randomly selected. The sample size was determined by the presumption that the data were sufficient based on the pretesting training sessions and pilot-testing results (78 posts) and consistency with previous sample sizes of Instagram studies [51,52]. This was later confirmed during the analysis by confidence in having reached, or closely approached, data saturation [53].

### Data Analysis

Since there was no codebook of AMR in general on SNSs, it was created based on previous analyses of AMR in SNSs and a scoping review of AMR communication. The creation of the codebook was based on the SMCR model, and the coding process was adapted from Cohen et al [52]. To enhance credibility and dependability, investigator triangulation measures were implemented. A stepwise approach alternating individual and team sessions was used to enhance intercoder consistency and reliability [54]. As a pretest, 4 coders independently analyzed 15 different posts each in training sessions, followed by an iterative process of consensus coding and updating the original codebook. In a successive pilot coding round, the 4 coders analyzed together a random sample of 18 posts in order to confirm consistency in categorization. The results of the pilot test were included in the final sample. Afterwards, each coder independently analyzed one-quarter of the posts. Each coder’s analysis was double-checked by another coder with full visibility of the original coding, and discrepancies were discussed and harmonized.

Content analysis was used in the study [55,56]. The unit of analysis comprised the caption, image, hashtags, and the content creator—their profiles were observed to categorize them according to their declared interests and visibility. Throughout the analysis, coders went back and forth between the post and the categories to enhance intracoder reliability and minimize the risk of misinterpretation [54]. During the coding process, posts were evaluated on the language used in captions, the emotional connotations of the images, and the context provided by hashtags. To assess the tone of the Instagram posts related to AMR, each post was categorized based on predefined sentiment categories: neutral, emotive, or promotional. For instance, posts categorized as emotive often included language that invoked fear or urgency about AMR, while neutral posts presented factual information without emotional framing. In the codebook, the application of the SMCR model generated 5 overarching categories, which are described together with the subcategories in Textbox 1. The categories in Textbox 1 were established through a rigorous content analysis of Instagram posts containing the hashtag “#antibioticresistance.” Each category reflects a distinct type of content creator, based on their users’ descriptions, bios, and linked websites. The professional background, communication style, and intended audience contributed to the categorization also. By categorizing content creators and their communication styles, this taxonomy provides valuable insights into the landscape of AMR discourse on Instagram, revealing both the strengths and limitations of current engagement strategies.

Textbox 1. **Description of the categories and subcategories.**

Sender: Content creator

Based on users’ descriptions, bios, and linked websites. The content of the post contributes to the categorization, too. The number of followers and “likes” per post was noted.

- Ecology and animal care: Users who connected antibiotic resistance (AR) to animal rights and animal care.
- Science and health: Users working in biomedicine, biotechnology, and medicine who connected AR to science and health.
- Information driver: Users who focus on spreading knowledge and raising awareness of AR.
- Naturopathist: Users who promote a holistic approach to wellness, often focused on lifestyle and diet.
- Pharmacy and veterinary: Users who connected AR to the pharmacy and veterinary fields.
- Education: Education institutions and students.
- Motivators and art: Wellness motivators and illustrators posting on AR.

Receiver: Audience

Inferred from the post content (image, caption, and hashtags) and considering the presumable interest of the content creator, that is, when the post creator caters or not specifically to their closest audience.

- General audience: The content does not appeal to any specific audience.
- Customers: The post, implicitly or explicitly, aims to sell products or services.
- Followers: The content is directed to the creator’s Instagram followers.
- Peers: The post is primarily directed to the creator’s peers, for example, vegan community and health care professionals.
- Patients: The post seeks to communicate with patients.
- Students: The post seeks to communicate with students.
- Local community: The post is directed to a local community.
- Health organizations: The post is directed toward health care organizations and policymakers.

Message: Purpose

- The manifest primary reason for which the post is made.
- Information and awareness: Spreading knowledge and raising awareness of AR.
- Advertising: Selling products or services.
- Propaganda: Promoting ideological or political points of view through biased or partial communication.
- Infotainment: Sharing humorous, relatable content, often through comics or illustrations to inform about AR.

Message: Tone

The gist or attitude of the post, including the image, caption, and hashtags.

- Neutral: A communication style tendentially tends to be information- and fact-based.
- Emotive: A communication style involving emotional content or potentially triggering an emotional response
- Promotional: A style involving marketing-style communication strategies

Message and channel: Centrality of AMR

An appraisal of AR importance within the post (image, caption, and hashtags).

- Main: AR is the most important subject in the post.
- Relevant: AR is an important subject in the post.
- Collateral: AR is a subject of secondary importance in the post.
- Irrelevant: The post is about something else, unrelated to AR (AR only appears in the hashtags).

In addition, other descriptive characteristics of the posts were noted: (1) the number of followers of the creator of the posts (sender); (2) the image content and type (message): text (the visual section of the post, whether static or a carousel, is dominated by words), visual object (the visual section of the post, whether static or a carousel, is dominated by a visual object, eg, images, pictures, photos, graphics, and illustrations), video, or audio; (3) the hashtags (channel); (4) the number of “likes” obtained by the posts (receiver).

### Ethical Considerations

This study analyzed publicly accessible Instagram posts tagged with “#antibioticresistance” using a custom web scraping script that accessed content viewable without login, mimicking human browsing behavior. The tool did not access private or password-protected data. While platform terms of service do not always clearly address noncommercial academic research, the study acknowledged the complex and evolving legal and ethical landscape of automated data collection [57]. No personally identifiable information was collected, stored, or quoted. Usernames, profile information, and other potentially identifying content were excluded to minimize the risk of reidentification. The posts included a mix of location-specific and nonlocation-specific content, and we did not systematically categorize them based on geographic references. The analysis focused on aggregated patterns in publicly shared communication, not on individual users.

Although ethical review was not required for the use of public data, the study followed established ethical guidance for social media research, including transparency in reporting, minimization of harm, and attention to user expectations of privacy [58,59]. The overall aim was to contribute to the understanding of public health communication, not to evaluate or profile individuals.

## Results

The results show that the sender categories could be divided into 7 main content creator types, with the biggest group being the information driver (n=186), followed by the professionals (n=147). Table 1 details the content creator types and general characteristics of their posts. The most common hashtags used are “bacteria,” “AMR,” “antimicrobialresistance,” “antibiotic,” “medicine,” and “health.” Typically, images contained text messages or pictures and illustrations—often of bacteria and test samples. The captions often described how antibiotic resistance (AR) and antimicrobial resistance (AMR) spread, why it needs to be stopped, and connected health consequences.

**Table 1.** Content creator types and general characteristics of their posts.

Category	Posts, n (%)	Top hashtags^a^ (n)	Top visual content^b^	Likes		Followers	
				Total^c^, n	Average^d^	Total^c^, n	Average^d^
Ecology and animal care	56 (9.2)	Vegan (13), plantbased (10), antibiotics (9), deforestation (7), factoryfarming (6), animalagriculture (5), eatmoreplants (5), animalagricultureisdestroyingtheplanet (4), biodiversityloss (4), eatplants (4), straightfromthefarm (4)	Food, meat, becoming vegan, and animals (cow and fish birds).	1566	28	329,578	5885
Science and health	147 (24.1)	Bacteria (27), AMR^e^ (25), microbiology (23), science (18), antibiotic (17), antimicrobialresistance (16), biology (13), medicine (12). Healthcare (11), lab (11), research (10)	Pills, bacteria, and lab tests.	25,440	173	3,315,184	22,552
Information driver	185 (30.3)	amr (92), antimicrobialresistance (79), superbugs (72), health (69), antibiotic (59), bacteria (59), medicine (57), stopsuperbugs (45), keepantibioticsworking (41), microbiology (37), antibioticstewardship (35), onehealth (35), science (35), healthcare (33), pharmacy(33), beantibioticsaware (30)	Pills, AMR facts, bacteria, doctor, and patient.	28,908	156	1,127,311	6094
Naturopathist	66 (10.8)	Antibiotics (29), health (12) antimicrobialresistance (11) bacteria (10), infection (8), beantibioticsaware (7), medicine (7), healthcare (5), healthy (5) wellness (5) microbiology (5) antibioticawareness (5)	Information, pills, hands, and animals.	3131	47	1,349,163	20,442
Pharmacy and veterinary	64 (10.5)	Antibiotics (24), antimicrobialresistance (15) pharmacy (14), antibiotic (13), antibioticstewardship (11), pharmacist (11) amr (10), bacteria (10), medical (7), doctor (7), infectiousdisease (7), resistance (7)	Dissertation, pills, bacteria, and hands.	1784	28	263,038	4110
Education	44 (7.2)	Antibiotics (10), bacteria (10), medicine (9), microbiology (8), amr (7), antibiotic (7) Science (7), antimicrobialresistance (6), publichealth (5), biology (4), pharmacy (4)	Research highlights, infographics research findings, and laboratory tests.	1269	29	326,894	7429
Motivators and art	48 (7.9)	Antibiotics (15), microbiology (11), bacteria (8), antibiotic (5), research (5) science (5), sciencecommunication (5) abstractart (4), art (4) artoftheday (4), podcast (4), ceramics (3)	Artwork, bacteria, selfies, and promoting hand hygiene products.	2970	62	45,771	954

a The top hashtags refer to the most frequent hashtags used per content creator category.

b The top image content refers to the most frequent pictures and illustrations used per content creator category.

c The total number of “likes” and “Followers”refers to the content creator categories, not to individual Instagram users.

d The average number of “likes” and “Followers”refers to the content creator categories, not to individual Instagram users.

e AMR: antimicrobial resistance.

In the following, each sender category (eg, “Ecology and animal care”) and their different sender subgroups (eg, “Vegans and vegetarians”) are described. For each sender category, the most relevant channel feature, namely the hashtags, and message features, such as characteristic visual content, are described. Accounting for the receiver, the number of “likes” is reported. For each sender subgroup, the emphasized message features include purpose, image content, tone, and centrality of AR. At the same time, as for the receiver analysis, the type of audience for which the post was made is shown.

### Ecology and Animal Care

These users connected AR to environmental and animal rights issues. The average number of “likes” per post (n=28) is relatively low in this sender category (see Table 1). Their top hashtags were “vegan,” “plantbased,” “antibiotics,” “deforestation,” and “factoryfarming.” Pictures often showed animals such as cows, fish, and birds and food products. The category was divided into 3 subgroups: “Vegans and vegetarians,” “Farming and food companies,” and “Others in food and diet.”

“Vegans and vegetarians” informed and promoted diet change and animal welfare to a great extent. The tone was emotive for most posts (56%, 18/32 participants) and, sometimes, they slipped into propaganda. Almost half of the posts (44%, 14/32 participants) have AR as a centrality in the post. Animal welfare was also crucial to the next subgroup—“Farming and food companies.” They encouraged eating organic and focused on showing good animal care and food production. Relatively often (14%, 3/21 participants) their posts served commercial purposes and were directed to their customers. Foremost, 10 out of 21 participants (48%) adopted a neutral tone in their posts, where AR was relevant but not the main focus of the post. These 2 subgroups are those with the highest number of posts where the centrality of AR was deemed only collateral (24%, 5/21 participants). In the “Others in food and diet” subgroup, organizations and individuals that share a focus on food and diet without falling into any other subgroups were grouped. Further details are shown in Table 2.

**Table 2.** Ecology and animal care subgroups’ characteristics.

Subgroup	Purpose, n (%)				Audience, n (%)			Image content, n (%)			Tone, n (%)			Centrality of AR^a^, n (%)			
	I&A^b^	Propaganda	Advertising	Infotainment	General	Peers	Customers	Text	Visual object	Video or audio	Emotive	Neutral	Promotional	Main	Relevant	Irrelevant	Collateral
Vegans and vegetarians (n=32)	25 (78)	4 (12.5)	3 (9.4)	0 (0)	28 (87.5)	4 (12.5)	0 (0)	18 (56.3)	11 (34.3)	3 (9.4)	18 (56)	10 (31)	4 (13)	14 (44)	7 (22)	6 (18)	5 (16)
Farming and food companies (n=21)	9 (43)	1 (4.8)	10 (47.6)	1 (4.8)	13 (61.9)	0 (0)	8 (38)	10 (47.6)	10 (47.6)	1 (4.8)	8 (38)	10 (48)	3 (14)	4 (19)	10 (48)	2 (10)	5 (24)
Others in food and diet (n=3)	2 (67)	0 (0)	1 (33.3)	0 (0)	3 (100)	0 (0)	0 (0)	2 (66.7)	1 (33.3)	0 (0)	1 (33)	1 (33)	1 (33)	1 (33)	2 (67)	0 (0)	0 (0)

a AR: antibiotic resistance.

b I&A: information and awareness.

### Science and Health

These sender categories showed their work within the AR field, either in research or care. Table 1 shows that the average number of “likes” per post (n=173) and users’ followers (n=22,552) was the highest among all main categories. The category is divided into 4 subgroups: “Biomedicine labs,” “Healthcare staff,” “Researchers,” and “Healthcare facilities.” Their top hashtags were “bacteria,” “AMR,” “microbiology,” “science,” and “antibiotic.” Pictures typically showed pills, laboratory tests, bacteria, and health care personnel.

“Biomedicine labs” encompasses collective profiles of laboratories and individuals creating laboratory-life content, thus focusing on scientific-specific aspects, such as AR mechanisms. This subgroup engages the most with its peers. The “Healthcare staff” subgroup consists of health care professionals. Their posts focused on proper antibiotic use and the threat posed by AR. Their audience was the public and, occasionally, a specific class of patients and their peers in health care. The “Researchers” subgroup comprises users who do research in academic or private settings. For the most part, they work in biomedicine and biotechnology. However, with respect to the “Biomedicine labs” subgroup, they focused more on individual work and achievements and, overall, gave a more personalized perspective to their posts and tried to reach a broader audience. The last subgroup, “Healthcare facilities,” involves hospitals, clinics, ambulatories, etc, often providing private services. Their user profiles are mainly collective or of individual users communicating on behalf of or about the health care facility. Their communication was similar to that of the “Healthcare staff” subgroup but tended to engage local communities and promote their services more. Further details are shown in Table 3.

**Table 3.** Science and health professionals subgroups’ characteristics.

Subgroups	Purpose, n (%)			Audience, n (%)							Image content, n (%)			Tone, n (%)			Centrality of AR^a^, n (%)			
	I&A^b^	Advertising	Propaganda	General	Peers	Health organization	Patients	Followers	Community	Students	Text	Visual object	Video or audio	Neutral	Emotive	Promotion	Main	Relevant	Collateral	Irrelevant
Biomedicine laboratories (n=37)	33 (89.2)	4 (10.8)	0 (0)	20 (54)	16 (43.2)	1 (2.7)	0 (0)	0 (0)	0 (0)	0 (0)	26 (70)	11 (29.7)	0 (0)	28 (76)	6 (16)	3 (8.1)	16 (43)	15 (41)	3 (8)	3 (8)
Health care staff (n=36)	31 (86.1)	3 (8.3)	2 (5.6)	28 (77.8)	5 (13.9)	0 (0)	3 (8.3)	0 (0)	0 (0)	0 (0)	17 (47.2)	18 (50)	1 (2.8)	26 (72)	5 (14)	5 (14)	25 (70)	5 (14)	4 (11)	2 (6)
Researchers (n=49)	41 (83.7)	4 (8.2)	4 (8.2)	40 (8.2)	8 (16.3)	0 (0)	0 (0)	1 (2)	0 (0)	0 (0)	19 (38.8)	26 (53)	4 (8.2)	37 (76)	9 (18)	3 (6)	31 (63)	15 (31)	2 (4)	1 (2)
Health care facilities (n=25)	20 (80)	5 (20)	0 (0)	15 (60)	0 (0)	0 (0)	3 (12)	0 (0)	5 (20)	2 (8)	4 (16)	18 (72)	3 (12)	15 (60)	6 (24)	4 (16)	16 (64)	5 (20)	3 (12)	1 (4)

a AR: antibiotic resistance.

b I&A: information andawareness.

### Information Driver

This main sender category of Instagram users is information-driven, and their manifest purpose is to spread knowledge and awareness. Their communication is effective, sometimes even entertaining, as humor is not disdained, and their content is tendentially short and easily accessible. As seen in Table 1, their top hashtags include “AMR,” “antimicrobialresistance,” “superbugs,” “health,” and “antibiotic.” Pictures depicted pills and health care personnel. They have a comparatively high engagement from their audience in terms of number of “likes” (156 on average per post). This category has 3 subgroups: “AMR organizations and communities,” “Journalists and reporters,” and “Companies.”

“AMR organizations and communities” involves nongovernmental organizations, communities, networks, and others focused on AMR. They mostly share information regarding AMR and AMR-related events (eg, the World AMR Awareness Week and conferences). This subgroup has the highest percentage of AMR-centered posts (84%, 66/79 participants), yet 14 out of 32 posts had a neutral tone (70%). The “Journalists and reporters” subgroup encompasses science news-based profiles, a few journals and newspapers or magazines, but also individual journalists and reporters. Usually, they reported recent scientific research results for the general audience but also for students and their followers and promoted AMR awareness and events. This subgroup tended more than others to use text in the image space of the posts (55 out of 77 participants). The last subgroup is “Companies.” They primarily used the platform to promote their products (eg, tests, drugs, and supplements) but delivered their communication in terms of AMR information and awareness spreading. They often targeted potential customers and health organizations. This subgroup tended more than others to use pictures in the image space of the posts (25 out of 30 posts). Further details are shown in Table 4.

**Table 4.** Information driver subgroups’ characteristics.

Subgroups	Purpose, n (%)				Audience, n (%)							Image content, n (%)			Tone, n (%)			Centrality of AR^a^, n (%)			
	I&A^b^	Advertising	Infotainment	Propaganda	General	Community	Patients	Students	Health organization	Followers	Customers	Visual object	Text	Audio or video	Neutral	Emotive	Promotion	Main	Relevant	Collateral	Irrelevant
AMR^c^ organizations and communities (n=79)	68 (86)	7 (8.8)	3 (3.8)	1 (1.3)	67 (84.8)	5 (6.3)	4 (5.1)	2 (2.5)	1 (1.3)	0 (0)	0 (0)	41 (51.9)	38 (48.1)	0 (0)	55 (70)	21 (27)	3 (4)	66 (84)	9 (12)	2 (3)	2 (3)
Journalists and reporters (n=77)	71 (92.2)	4 (5.2)	2 (2.6)	0 (0)	55 (71.4)	0 (0)	0 (0)	9 (11.7)	2 (2.6)	8 (10.4)	3 (3.9)	21 (27.3)	55 (71.4)	1 (1.3)	48 (62)	21 (27)	8 (10)	48 (62)	14 (18)	10 (13)	5 (7)
Companies (n=30)	18 (60)	9 (30)	1 (3.3)	2 (6.7)	17 (56.7)	0 (0)	0 (0)	0 (0)	7 (23.3)	0 (0)	6 (20)	25 (83.3)	5 (16.7)	0 (0)	23 (76)	4 (14)	3 (10)	19 (62)	7 (24)	1 (3)	3 (10)

a AR: antibiotic resistance.

b I&A: information and awareness.

c AMR: antimicrobial resistance.

### Naturopathist

These users promote a holistic approach to wellness, often focused on lifestyle and diet. An active lifestyle, keeping a balanced gut microbiota through wise food choices, and avoiding unnecessary antibiotic use were hot topics. The most common hashtags were “antibiotics,” “health,” “antimicrobialresistance,” “bacteria,” and “infection.” Pictures were often associated with food or health. This sender category includes the subgroups: “Natural healers,” “Body and nutrition,” and “Environment and health” subgroups.

Specific to the “Natural healers” was the latent message that the individual users were personally engaged in AMR. Relatively often, the posts were used to promote their products (eg, probiotics and natural antimicrobials) or services (eg, mindfulness and homeopathy consultancies). The second subgroup, “Body and nutrition,” included fitness enthusiasts who share information and tips about healthy eating. The “Environment and healthcare” subgroup involves individuals focusing on the environment, health, and sustainability. The latter two subgroups’ tone was coded as the most emotive with 3 out of 5 and 2 out of 4 posts being emotive. The last group’s communication was even characterized by anger. Further details are shown in Table 5.

**Table 5.** Naturopathist subgroups’ characteristics.

Subgroups	Purpose, n (%)				Audience, n (%)						Image content, n (%)			Tone, n (%)			Centrality of AR^a^, n (%)			
	I&A^b^	Advertising	Propaganda	Infotainment	General	Customers	Followers	Health organization	Patients	Community	Text	Visual object	Video or audio	Neutral	Emotive	Promotion	Main	Relevant	Collateral	Irrelevant
Natural healers (n=57)	43 (75.4)	10 (17.5)	3 (5.3)	1 (1.8)	43 (75.4)	9 (15.8)	3 (5.3)	1 (1.8)	1 (1.8)	0 (0)	29 (50.9)	27 (47.4)	1 (1.8)	41 (72)	11 (19)	5 (9)	29 (51)	16 (28)	7 (12)	5 (9)
Body and nutrition (n=5)	4 (80)	1 (20)	0 (0)	0 (0)	4 (80)	0 (0)	0 (0)	0 (0)	0 (0)	1 (20)	2 (40)	3 (60)	0 (0)	2 (4)	3 (60)	0 (0)	4 (80)	0 (0)	1 (20)	0 (0)
Environment and health (n=4)	3 (75)	1 (25)	0 (0)	0 (0)	3 (75)	1 (25)	0 (0)	0 (0)	0 (0)	0 (0)	4 (100)	0 (0)	0 (0)	1 (25)	2 (50)	1 (25)	2 (50)	0 (0)	2 (50)	0 (0)

a AR: antibiotic resistance.

b I&A: information and awareness.

### Pharmacy and Veterinary

Users in this sender category focus on spreading knowledge about correct antibiotic use and AR and promoting their products and services for human and animal health. More than other categories, they include “One Health” in their posts. Their top hashtags involve “antibiotics,” “antimicrobialresistance,” “pharmacy,” “antibiotic,” and “antibioticstewardship,” and the pictures primarily concern pills, bacteria, and hands (see Table 1).

The subgroups are “Pharmacists and vets”—pharmacy and veterinary medicine professionals—and “Pharmacy and veterinary companies.” The latter subgroup has the highest promotional tone with 4 out of 15 of the posts (27%). Further details are shown in Table 6.

**Table 6.** Pharmacy and veterinary subgroups’ characteristics.

Subgroups	Purpose			Audience				Image content			Tone			Centrality of AR^a^			
	I&A^b^	Infotainment	Advertising	General	Peers	Customers	Health organization	Text	Visual object	Video or audio	Neutral	Emotive	Promotion	Main	Relevant	Irrelevant	Collateral
Pharmacists and vets (n=49)	43 (87.8)	5 (10.2)	1 (2)	38 (77.6)	6 (12.2)	4 (8.2)	1 (2)	30 (61.2)	18 (36.7)	1 (2)	32 (65)	14 (29)	3 (6)	39 (80)	6 (12)	3 (6)	1 (2)
Pharmacy and veterinary companies (n=15)	9 (60)	0 (0)	6 (40)	9 (60)	0 (0)	3 (20)	3 (20)	8 (53.3)	7 (46.7)	0 (0)	9 (60)	2 (13)	4 (27)	8 (53)	4 (27)	2 (13)	1 (7)

a AR: antibiotic resistance.

b I&A: information and awareness.

### Education

This sender category top hashtags were “antibiotics,” “bacteria,” “medicine,” “microbiology,” and “AMR.” Their top images comprehended research highlights, infographics, research findings, and laboratory tests. The category encompasses “Students” and “Education institutes.” The subgroup “Students” includes upper secondary education students, undergraduates, and graduate students (PhD students were placed in the Science and Health category).

Users in the “Students” subgroup shared what they learned about AR and promoted proper antibiotic use. Also, users in “Education institutes” created posts to spread awareness, besides promoting their courses and activities and highlighting research findings. It is the category whose posts were coded as using the most neutral tone (91%, 21/23 posts). Further details are shown in Table 7.

**Table 7.** Education subgroups’ characteristics.

Subgroups	Purpose			Audience			Image content		Centrality of AR^a^			
	I&A^b^	Infotainment	Advertising	General	Peers	Students	Visual object	Text	Main	Irrelevant	Relevant	Collateral
Students (n=21)	19 (90.5)	2 (9.5)	0 (0)	17 (80.9)	4 (19)	0 (0)	16 (76.2)	5 (23.8)	15 (71)	3 (14)	2 (10)	1 (5)
Educational institutes (n=23)	18 (78.3)	0 (0)	5 (21.7)	18 (78.3)	0 (0)	5 (21.7)	14 (60.9)	9 (39.1)	15 (65)	0 (0)	7 (30)	1 (4)

a AR: antibiotic resistance.

b I&A: information and awareness.

### Motivators and Art

This sender category’s top hashtags were “antibiotics,” “microbiology,” “bacteria,” “antibiotic,” and “research.” Their top pictures included artwork, bacteria, and selfies (see Table 1). The category involves users who are less focused on AMR. Its subgroups are “Motivators” and “Art.” The former involves users who try to influence and provide feed content of interest, including AR. The latter involves illustrators and art communities that have created content for AR.

The “Motivators” subgroup often resorted to personal narratives, connected AR to life experiences, and promoted healthy habits. The “Art” subgroup used relatively more pictures in the image space of the posts, and 4 out of 13 (31%) used an emotive (sad or scary) tone. Further details are shown in Table 8.

**Table 8.** Motivators and art subgroups’ characteristics.

Subgroups	Purpose				Audience		Image content			Tone			Centrality of AR^a^			
	I&A^b^	Infotainment	Advertising	Propaganda	General	Followers	Visual object	Text	Video or audio	Neutral	Emotive	Promotion	Main	Relevant	Irrelevant	Collateral
Motivators (n=35)	30 (85.7)	3 (8.6)	2 (5.7)	0 (0)	31 (88.6)	4 (11.4)	21 (60)	11 (31.4)	3 (8.6)	26 (74)	8 (23)	1 (3)	19 (54)	9 (26)	4 (11)	3 (9)
Art (n=13)	10 (77)	1 (7.7)	1 (7.7)	1 (7.7)	12 (92.3)	1 (7.7)	9 (69.2)	4 (30.8)	0 (0)	9 (69)	4 (31)	0 (0)	5 (38)	6 (46)	1 (8)	1 (8)

a AR: antibiotic resistance.

b I&A: information and awareness.

## Discussion

### Principal Findings

The main findings of this study reveal that while Instagram serves as a platform for disseminating accurate and informative content about AMR, the discussions are largely confined to niche groups, limiting broader audience engagement. The findings showed a heterogeneous and siloed user landscape, where top-down communication is delivered by those who are knowledgeable or have a linked purpose, often of a commercial nature with minimal interaction beyond specific interest communities, suggesting a need for more inclusive and visually compelling content to enhance public awareness and engagement on this critical health issue. These results confirm previous studies [24-26].

### Senders and Receivers

Although users created posts that were accessible to a general audience, the broader public rarely engaged with the content or showed visible interest. Virtually no content was created by someone not already engaged in the AMR field or not promoting specific viewpoints, services, or goods. The fragmented and siloed landscape of AMR on Instagram is also confirmed in the analysis of user engagement patterns, which reveals that “likes” of posts and interactions occur predominantly within specific user groups, with each subgroup gravitating towards content tailored to their interests and priorities. This is shown in the images, the way posts are formulated and that most “likes” derive from other users within the same main category. For instance, a post by a “Science and health” user related to AMR and infection prevention would gain higher engagement rates among users in the same category and individuals with a vested interest in public health issues. In a nutshell, AMR is of no interest to the Average Joe of Instagram. Only a few posts had health organizations among their recipients and very few health authorities and policymakers, highlighting the platform’s limitations as a forum for broader policy discussion. The challenge of informational homophily and its siloing effect for which science and health content reach almost exclusively already engaged audiences has been long known, and it was also detected in this study, indicating challenges in overcoming audience segmentation on SNSs [60,61]. It is also possible that algorithmic filtering limits the visibility of AMR content to users outside niche communities. Since content exposure is shaped by previous behavior and engagement patterns, users who are not already interacting with health-related content may be less likely to encounter posts about AMR, even if they might find the topic relevant or engaging [62].

The major actors in the AMR discussion on Instagram are the “Science and health” and “Information driver” content creators. These 2 groups are highly engaged in the AMR field, either possessing extensive professional expertise or being well-informed on the subject. Both groups want to raise awareness, but they do it either by practicing stewardship AMR (Science and health) or mobilizing individual and societal actions (Information drivers). They, together with “Pharmacy and veterinary” and “Education” users, have a specific responsibility deriving from being (and being perceived as) specialists and experts to communicate about AMR in a truthful, nonbiased, and educated manner, as whenever they engage in health and science communication through SNSs, there is the potential for impacting public health and individual behavior and public trust in general [63,64]. These senders are perceived as experts, and their social status as professionals or well-informed individuals lends authority to their messages. This aligns with the SMCR model, where the sender’s characteristics—like their knowledge and credibility—determine the effectiveness of communication.

### Messages

The purpose of 3% of the posts (18/611) was coded as propaganda, meaning that the content promoted ideological or political points of view through biased or partial communication. For the majority, this type of communication was adopted by users who stressed their concern for animal health and the environmental consequences of AMR. Therefore, AMR and antibiotic use are not ideologically or politically invested in themselves, at least not yet. Such communication happened in conjunction with animal and environmental themes, which tend instead to be polarizing, especially in social media, which are designed to monetize on disagreement among users [65].

Overall, the content of the posts was accurate and conveyed in a neutral tone. Narratives and images aimed at triggering an emotional response were also used but without compromising the veracity of the content, thus to be considered, for the most part, as a way to attract an audience and motivate behavior change through the mediating role of self-relevant emotions (primarily fear) [66]. Noteworthily, almost no content highlighted the role and responsibilities of institutions about AMR.

Posts were text-dominated to an extent above average for Instagram users. This was predictable, considering that they aimed at spreading information and raising awareness about AMR. A 2020 study on the Centers for Disease Control and Prevention’s Instagram posts showed similar results about the role of text in the visual section of the analyzed posts [67]. A recent research study by Charani et al [68] on the message content produced by key actors in global health about the visual depiction of AMR found that the current narrative is one of power imbalances, where women and children from low-income and middle-income countries are presented with less dignity, respect, and power than those from high-income countries. On a positive note, none of this was detected in this study. Therefore, the problem of degradingly representing AMR stands with international health bodies but is not shared by other Instagram content creators, not even in the posts whose purpose was deemed as propaganda or that adopted an emotive tone. Charani et al [68] has also shown that imagery in global health communication plays a crucial role not just in transmitting facts but in shaping public understanding, emotional responses, and awareness of infectious diseases, including AMR. Their analysis of visual practices across global health documents highlights how images, when ethically and contextually used, can embed health issues in the public imagination and evoke empathy [68]. In our study, many Instagram posts featured images such as stylized bacteria, pills, or laboratory scenes. However, these visuals were often used in an illustrative or aesthetic way, with limited contextual or emotional framing. Given Instagram’s visual-first design, future AMR communication on the platform could benefit from adopting more purposeful and ethically grounded imagery to broaden public engagement and improve risk communication.

### Channels

In this study, hashtags are considered as channels, serving multiple functions beyond grouping content. Hashtags, in this context, were also a vehicle for marketing, activism, and community engagement, allowing senders to align their messages with specific networks of associations and broadening the scope and impact of their communication. Different sender categories used hashtags strategically based on their intended message and audience. For example, users in the “Ecology and animal care” category used hashtags like “vegan,” “plantbased,” and “deforestation” to align AR with environmental and animal rights issues. Here, hashtags extended the message beyond health care, invoking broader themes that resonate with specific “green” values and networks. Similarly, users in the “Science and health” category frequently used hashtags such as “bacteria,” “AMR,” and “microbiology,” which are directly related to scientific work and communication. These hashtags helped cater scientific content to targeted peers within the scientific community, as well as an informed public. Hashtags also served a commercial function, particularly in the “Pharmacy and veterinary” and “Information driver” categories. Hashtags like “pharmacy,” “antibioticstewardship,” and “health” not only connected the posts to public health but were also marketing tools for promoting products and services, often with a neutral tone to maintain credibility.

Rauschnabel et al [69] identified 10 different motivations for using hashtags (in order of frequency): amusing, organizing, designing, conforming, trendgaging, bonding, inspiring, reaching, summarizing, and endorsing. This research mainly showed organizing and reaching use, namely, to structure and organize the content of posting and to meet the conventions of specific groups of interest, respectively. To a minor extent, also conforming to use, that is, showing the desire to meet the conventions of specific groups of interest was observed.

### Limitations

The study findings should be interpreted considering several methodological choices. A key strength lies in the use of stratified random sampling based on time, which ensured a balanced representation of content across the 24-month period while maintaining analytic feasibility. A 10% sample was drawn (N=610), consistent with established practices in media and communication research where full-population analysis is often impractical [70]. While this enabled the identification of dominant patterns and themes, rare or emerging discourses may have been underrepresented. Temporal stratification helped mitigate this by capturing variation in posting behavior over time [71]. To enhance credibility and dependability, coding was conducted iteratively, including pilot testing, coder training, and cross-checking. These strategies, along with attention to data saturation, supported the trustworthiness of findings. The study focused exclusively on Instagram and the hashtag “#antibioticresistance.” While this defined a clear and relevant dataset, AMR-related discussions may also occur under other hashtags or on other SNSs such as X or Facebook. Platform-specific user bases and engagement formats may influence how AMR is communicated and perceived. Future studies should broaden the scope to include additional platforms and terms. Automated accounts were not identified or excluded in this study. Since bots can inflate engagement metrics, future work should consider detection methods to ensure accurate interpretations of user interaction.

Finally, while the SMCR model provided a useful structure, it does not fully capture the participatory, multimodal nature of SNSs. Future work may benefit from combining it with frameworks better suited to networked communication.

### Conclusions

The findings of this study provide an overview of how AMR is communicated on Instagram, particularly through the lens of the hashtag “#antibioticresistance.” It is evident that while Instagram serves as a platform for disseminating valuable information about AMR, the engagement with this content is largely confined to niche communities rather than reaching a broader audience. This conclusion synthesizes the key findings, discusses the implications for public health communication, and suggests pathways for enhancing engagement and awareness around AMR on social media platforms.

The content analysis of 611 Instagram posts revealed several critical insights into the landscape of AMR communication. First and foremost, the primary intention behind these posts was to inform the public about AMR, with 497 out of 611 of the posts (81.3%) categorized as information-driven. The predominant content creators were classified as “Information drivers” and “Science and health” professionals, who are engaged in raising awareness about AMR. Their posts were characterized by a neutral tone, with a significant amount of text-based content aimed at educating the audience about the implications of antibiotic resistance.

Despite the accuracy and neutrality of the content being commendable, the study highlighted a concerning trend of the isolated nature of the conversations of AMR within specific interest groups. The majority of engagement, as indicated by the number of likes and interactions, occurred within these niche communities, suggesting that the broader Instagram audience, or average individuals, remains largely disengaged from AMR discourse. This finding aligns with existing research that emphasizes the challenges of reaching diverse audiences in health communication, particularly in the context of social media.

The implications of these findings for public health communication are significant. AMR represents a pressing global health crisis, and effective communication strategies are essential for raising awareness and fostering behavioral change among the general population. Given the reliance on social media as a primary source of information for many individuals, particularly younger demographics, it is critical that AMR discussions are made more accessible and engaging. The findings of this study highlight the need for more inclusive, relatable, and visually engaging content that can resonate with a broader audience. To address the challenges identified in this study and promote communication that is actionable and impactful for diverse audiences, the following recommendations are proposed: (1) integrate AMR discussions into more general interest posts, such as stories related to daily life, popular culture, and trending topics in order to attract the attention of nonexperts; (2) use more visually engaging content that cuts through the noise; (3) increase institutional engagement; (4) promote inclusive narratives; (5) improve engagement strategies and campaigns that require user participation, such as challenges, question-and-answer sessions, and live discussions, to boost engagement and interaction or series of educational posts; and (6) highlight the individual and collective responsibility for AMR. Ultimately, addressing the challenges of siloing and promoting a more inclusive dialogue will be essential in mobilizing collective action against this critical global health issue.

## Abbreviations

AMR: antimicrobial resistance
SMCR: sender-message-channel-receiver
SNS: social network site
WAAW: World Antimicrobial Resistance Awareness Week
WHO: World Health Organization
